# Hexokinase 2 Suppression Alleviates the Catabolic Properties in Osteoarthritis via HMGA2 and Contributes to Pulsed Electromagnetic Field-mediated Cartilage Protection

**DOI:** 10.7150/ijbs.101597

**Published:** 2025-01-27

**Authors:** Chuncha Bao, Siyi Zhu, Dejiang Pang, Ming Yang, Jiapeng Huang, Fengsheng Wang, Yue Hou, Xiangxiu Wang, Yuan Feng, Haolun Yang, Junliang Jiang, Jing He, Chengqi He

**Affiliations:** 1 Department of Rehabilitation Medicine, West China Hospital, Sichuan University, Chengdu, 610041, Sichuan, People's Republic of China.; 2 Key Laboratory of Rehabilitation Medicine, West China Hospital, Sichuan University, Chengdu, People's Republic of China.; 3 Department of Neurology, Laboratory of Neurodegenerative Disorders, National Clinical Research Center for Geriatrics, West China Hospital, Sichuan University, No.37, Guoxue Lane, Chengdu, Sichuan, 610041, China.; 4 The Lab of Aging Research, State Key Laboratory of Biotherapy, National Clinical Research Center for Geriatrics, West China Hospital, Sichuan University, Chengdu, China.; 5 Clinical Medical College of Acupuncture-Moxibustion and Rehabilitation, Guangzhou University of Chinese Medicine, Guangzhou, Guangdong 510006, PR China.; 6 State Key Laboratory of NBC Protection for Civilian, Beijing 102205, China.

**Keywords:** Osteoarthritis, Pulsed electromagnetic field, Hexokinase 2, Cartilage damage

## Abstract

Abnormalities in glycolytic pathways are prominent factors in the pathogenesis of osteoarthritis (OA). The key glycolytic enzyme Hexokinase 2 (HK2) is highly expressed in chondrocytes in OA; however, its role remains unclear. Pulsed electromagnetic field (PEMF) is commonly used for the treatment of OA. However, the role of PEMF in cartilage damage and the underlying mechanisms are not well understood. Herein, we found that HK2 suppression down-regulated catabolic pathways and alleviated inflammatory responses in OA chondrocytes, whereas HK2 overexpression stimulated inflammation and catabolic levels; moreover, inhibition of HK2 has potential anti-inflammatory and anti-catabolic properties by regulating the expression of HMGA2. PEMF dramatically inhibited the increase in glycolytic activity and catabolic metabolism level in OA and could alleviate the OA phenotype by modulating the HK2/HMGA2 signaling axis. Suppressing HK2 via adeno-associated virus (AAV) in articular cartilage demonstrated that PEMF reduces cartilage damage and OA symptoms through HK2 knockdown. Furthermore, the HK2 inhibitor Lonidamine, in combination with PEMF, more effectively ameliorated cartilage degeneration in OA. Overall, our findings improve understanding of HK2's role in OA and offer new insights for targeting HK2 in treatment. Furthermore, our results provide new clues for the reducing of catabolism and cartilage damage using PEMF.

## Introduction

Osteoarthritis (OA) is the most common chronic degenerative and disabling joint disease of the musculoskeletal system 1, 2. Cartilages are one of the important components of the knee joint, and chondrocytes maintain the integrity of cartilages through the homeostasis of the extracellular matrix (ECM) 3. The pathogenesis of OA is relatively complex, involving genetic, metabolic, and biochemical factors 4. Current evidence suggests that metabolic dysregulation plays various regulatory roles in the pathogenesis of OA, including mitochondrial metabolism, glycolysis metabolic pathways, the tricarboxylic acid cycle (TCA), lipid metabolism, and amino acid metabolism 5-8. In the OA environment and under inflammatory stimulations, chondrocytes undergo pathological changes in metabolic homeostasis and cartilage remodeling 9. In addition, metabolic abnormalities in chondrocytes can lead to an imbalance in collagen synthesis and degradation, cartilage degeneration, and ultimately result in the onset of OA 10. Additionally, metabolic abnormalities increase susceptibility to OA, and also impede the functional recovery of patients following joint replacement surgery 7. Results from cross-sectional clinical studies have indicated a significant positive correlation between the severity of symptomatic Knee Osteoarthritis (KOA) and the metabolic syndrome accumulation factor 11. Therefore, a comprehensive understanding of the complex mechanisms underlying the association between metabolic abnormalities and OA may lead to the development of new therapeutic strategies.

Glycolysis is the process of breaking down glucose to produce pyruvate. It occurs in the cytoplasm and is one of the most important pathways for glucose metabolism in the body 12. Abnormalities in the glycolytic metabolic pathway can lead to chondrocyte hypertrophy and ECM degradation in chondrocytes 13. Recent studies indicated that after stimulating chondrocytes using inflammatory factors, there was an upregulation of key glycolytic genes and an enhancement of glycolytic activity 14. Hexokinase 2 (HK2), the first rate-limiting enzyme in glycolysis, converts glucose to glucose-6-phosphate (G-6-P) 15. Studies have demonstrated that HK2 is highly expressed in fibroblast-like synovial cells (FLS) in OA, and overexpression of HK2 in chondrocytes could promote the secretion of inflammatory factors, including IL-6 and IL-8 16. However, although HK2 may be involved in the pathogenesis of OA, the specific mechanism underlying the involvement of HK2 in the progression of OA remains unclear.

High-mobility group AT-hook 2 (HMGA2) is an architectural transcription factor that directly binds to DNA sequences 17-19. HMGA2 has been shown to exhibit diverse biological functions and contribute to angiogenesis, tumor metastasis, self-renewal of hematopoietic stem cells and neuronal stem cells, and OA cartilage synthesis 19-22. A recent study demonstrated that HMGA2 activates Sox9 transcription by binding to regulatory sequences and promoters in chondrocytes. Furthermore, SOX9 promotes the expression levels of synthetic metabolic genes, such as COL2A1 and aggrecan. However, the role of HMGA2 in the pathogenesis of OA mediated by HK2 remains unclear. Therefore, it is crucial to clarify whether HK2 affects the pathogenesis of OA by regulating HMGA2, especially in promoting the potential mechanisms of chondrocyte synthesis and catabolism 19.

Due to the complex pathogenesis of OA, current clinical treatment methods for OA remain limited. Non-steroidal anti-inflammatory drugs (NSAIDs) and other analgesics only temporarily alleviate symptoms; furthermore, excessive use of these drugs can lead to hepatic and renal damage. Moreover, these drugs cannot reverse the progression of OA 23. Pulse electromagnetic field (PEMF) is a non-invasive, painless, cost-effective, and safe form of biophysical stimulation that can regulate metabolic processes such as oxidative stress 24, 25, mitochondrial dysfunction, and energy metabolism 26, thus offering therapeutic effects against a variety of diseases. Both clinical trials and preclinical studies have shown that PEMF can reduce inflammatory responses and enhance subchondral bone sclerosis, thereby ameliorating the progression of OA 27, 28. However, the mechanisms by which PEMF exerts its therapeutic effects on OA are unclear. Therefore, elucidating the mechanisms of PEMF treatment for OA will aid in its widespread adoption in clinical practice.

In this study, we found that the key glycolytic kinase, HK2, is significantly increased in the damaged human cartilages in OA and in IL-1β-induced primary chondrocytes from mice, with higher expression levels observed in the damaged regions compared to the undamaged areas. This study aims to investigate the role of HK2, a key glycolytic kinase, in the progression of OA and its potential as a therapeutic target. Specifically, we explore the expression patterns of HK2 in damaged human cartilage and IL-1β-induced primary chondrocytes, evaluate the involvement of the HK2/HMGA2 signaling pathway in OA-related catabolic processes, and assess the therapeutic effects of PEMF intervention. Additionally, we examine the combined effects of PEMF and the HK2 inhibitor Lonidamine on cartilage metabolism to identify strategies for balancing catabolic and anabolic processes in OA. This study seeks to provide a foundation for innovative approaches to OA treatment by targeting HK2.

## Materials and Methods

### Cell culture

Mouse primary chondrocytes were isolated from 5-7-day-old male C57BL/6 J mice. After the mice were sacrificed, they were disinfected using 75% alcohol. Afterwards, articular cartilage samples were removed, rinsed with normal saline, and then sectioned using a pair of scissors. These samples were subjected to a digestion buffer (consisting of DMEM/F-12 supplemented with collagenase II) and incubated at 37 °C overnight. The following day, the digestion buffer was filtered and centrifuged to obtain primary chondrocytes. The isolated chondrocytes were cultured in DMEM/F-12 (Gibco, USA) medium containing 10% FBS and 100 IU/mL of penicillin/streptomycin in an incubator with 5% CO₂ at 37 °C. ATDC5 cells (iCell Bioscience Inc, China) were cultured in DMEM/F12 (Gibco, USA) containing 10% FBS (Gibco, USA), 100 units/mL of penicillin, and streptomycin (Gibco, USA) and incubated under standard conditions (37 °C, 5% CO_2_). We incubated primary chondrocytes with IL-1β (10 ng/mL) for 24 h to simulate OA chondrocytes *in vitro*.

### Animals and surgery

C57/BL mice (12 weeks old, weight 20-25 g) were used. All animal studies were approved and performed by the animal Care and Use Committee of West China Hospital of Sichuan University (20220224015). Mice were placed in identical specific pathogen-free (SPF) standard environmental conditions (with 5 animals per cage, at 23 ± 2°C, and a 12-h light/dark cycle). The mice were allowed to move freely in the cages with access to food and water ad libitum. The destabilization of the medial meniscus (DMM) and sham surgery were performed as previously reported 29, mice in the sham surgery group served as controls (n=8 per group). After DMM modeling, the mice were randomly divided into groups for intervention. The control group maintained consistent feeding conditions except for those without intervention. OARSI and micro-CT were used to evaluate whether the modeling was successful. According to the preliminary experiment, when the sample size *n* is ≥ 4, *a* is 0.05 and the power is 95%. Mice with unsuccessful modeling and those that had natural death were excluded. The PEMF instrument setup was developed and provided by the School of Manufacturing Science and Engineering, Sichuan University, with two sets of equipment (for animal intervention and cell intervention). In this study, an output pulsed square signal of 75 Hz and 1.6 mT were determined based on previous research by our research group (Intervention for 60 minutes each time, once a day, were treated for 30 days).

### Human OA specimens

The human articular cartilage specimens were taken from patients who underwent total knee replacement surgery at West China Hospital of Sichuan University, and were divided into relatively healthy (Undamaged, non-OA) and severely injured (Damaged, OA) areas. Informed consent was obtained for sample collection according to the protocols approved by the institutional review board of the West China Hospital of Sichuan University and in accordance with the principles of the Declaration of Helsinki.

### qRT-PCR

Total RNA was isolated using RNAiso Plus (Takara, Japan) and reverse transcribed into cDNA using 5X RT Master Mix (Takara, Yeasen, China). Quantitative PCR was performed using the QuantStudio 3 Real-Time PCR system with SYBR Green PCR Master Mix (Thermo Fisher Scientific, USA). The PCR primers (Primer sequences) are shown in Supplementary Table 1). The relative quantification of gene expression was calculated with a 2-ΔΔCT method, and normalized to the GAPDH expression level.

### Cell transfection

Primary chondrocytes were transfected using HK2siRNA (Tsingke Biotechnology, Shanghai, China), the target plasmid pcDNA3.1 (+)-Myc HMGA2 HisA (Gene Pharma, Suzhou, China), and the siRNA+NC and pcDNA3.1(+) myc-HisA as controls, using jetPRIME® transfection reagent (Invitrogen) according to the protocol of the manufacturer. To exclude any siRNA off-target effect, three sequences were used. The lnc-NR3C siRNA target sequences were as follows: siRNA-1, 5′- GCGAGAUUGGUCUCAUUGUTT′; siRNA-2, 5′- CGAAUCUGCCAGAUUGUGUTT-3′; siRNA-3, 5′- GUAGAGAUGCAUAACAAGATT-3′, and NC, 5′-UUCUCCGAACGUGUCACG UTT-3′. In our study, these three kinds of siRNAs were mixed (siRNA pool) to achieve the interference effect while eliminating the off-target effect by reducing the use of each kind of siRNA.

### Lentivirus transfection

The Lv-HK2 and Lv-NC lentiviruses were purchased from Gene Pharma (Shanghai, China). The cells were transfected at 70-80% confluency. After 48 h, the medium was changed and puromycin (2.5 µg/mL) was added to filter stable transgenic strains of the target gene overexpression lentivirus.

### Enzyme activity assay

The biological activities of lactic dehydrogenase (LDH), Pyruvate kinase (PKM) and HK (solarbio, Beijing, China) in cell samples and serum samples were tested according to the manufacturer's protocols of kits. The lactic acid (LA) level (solarbio, Beijing, China) was tested according to the protocols described by the manufacturer of the testing kits.

### Cell bioenergy tests

Chondrocytes were seeded in XFe 96-well microplates (5000 cells/well) (Agilent Technologies, USA). We used the Agilent Seahorse XFp mitochondrial oxygen consumption rate assay kit and extracellular acidification rate assay kit (Agilent Technologies, USA) to assess cellular energy metabolism. The Hippocampal Cell Energy Metabolizer was also used to measure the oxygen consumption rate (OCR) and extracellular acidification rate (ECAR) of chondrocytes. Data was measured using the Seahorse Wave 2.6 software to analyze experimental results.

### Micro-CT analysis

The knee joints of mice were harvested and fixed in 4% paraformaldehyde fix solution for 48 h. The knee joints were then scanned using a micro-CT instrument (Quantumnbsp; GX, PerkinElmer, USA). We set the scanner at a resolution of 50 μm with voltage, 80 kV; and electric current, 100 μA. The resulting scanning profiles were further analyzed using Imaris 9.9 software and Mimics Inovation Suite 21 software to obtain three-dimensional reconstruction images of knee joints. The caliper analyze 12.0 software was used to analyze the trabecular bone/volume per total volume (BV/TV) score.

### Histological observation

Knee joint samples were decalcified in 14% EDTA, then the tissues were embedded in paraffin and cut to a thickness of 5 μm. The sections were processed using hematoxylin & eosin (H&E) staining, toluidine blue staining, and safranin O-fast green staining. The osteoarthritis research society International (OARSI) was used to estimate the degree of cartilage degradation; the scores were from 0 to 6. Immunohistochemical staining was performed using sections. We detected the protein expression of COL2A1 (Alpha-1 type II collagen, Abcam, USA; 1:500), Aggrecan (Abcam, USA; 1:500), MMP3 (Matrix Metallo proteinase 3; HUABIO, China; 1:200, MMP13 (Matrix Metallo proteinase 13, HUABIO, China; 1:200), and HK2 (ImmunoWay, USA; 1:200). The next day, after incubation with the secondary antibody for 1 h at room temperature, samples were stained with DAB, as well as hematoxylin. The staining result of the samples were observed using an optical microscope (Carl Zeiss, Germany).

### Immunofluorescence staining

The knee joint samples and chondrocytes were incubated with primary antibodies COL2A1 (Abcam, USA; 1:500), aggrecan (Abcam, USA; 1:500), MMP3 (HUABIO, China; 1:200, MMP13 (HUABIO, China; 1:200), and HK2 (ImmunoWay, USA;1:200) at 4 ℃ overnight. Afterwards, secondary antibodies (fluorescent Alexa Fluor® 488 goat anti-rabbit and fluorescent Alexa Fluor® 568 goat anti-rabbit) were applied at 37 °C in the dark for 1 h. Fluorescence images were captured using a Nikon fluorescence microscope.

### RNA sequencing assays

RNA samples were extracted in the OA group and the OA+HK2siRNA group, and utilized for RNA sequencing to verify the changes in mRNA expression profiles. Differently expressed genes between the OA group and OA+HK2siRNA group were screened using DE Seq software (1.20.0). Furthermore, topGO was used to perform GO enrichment analysis on the differential genes. The KEGG signal pathway enrichment analysis was performed using ClusterProfiler (3.4.4) online software. The statistical significance of the fold differences in RNA levels between the OA group and the OA+HK2siRNA group was determined using a two-tailed paired t test, with a P value of <0.05 was considered significant.

### Western blotting assay

After cell treatment, the chondrocytes were added to the RIPA buffer (Beyotime, Beijing, China) mixing with protease/phosphatase inhibitor (Beyotime, Beijing, China). Additionally, the BCA protein assay reagent (Beyotime, Beijing, China) was used to measure the protein concentration. Afterwards, 10% SDS-PAGE was applied to separate the purified proteins (30-50 μg). The gel was transferred to PVDF membranes and blocked with 5% non-fat milk (Beyotime, Beijing, China). The membranes were incubated with the corresponding primary antibodies overnight at 4 °C. The following primary antibodies were used: MMP13 (HUABIO, China; 1:1000), HK2 (ImmunoWay, USA; 1:1000), HMGA2 (ImmunoWay, USA; 1:1000), and ADAMTS5 (Abcam, USA, 1:1000). The membranes were incubated using horseradish peroxidase (HRP)-conjugated secondary antibodies (HUABIO, China, 1:5000) and developed with the chemiluminescence imager (Bio-Rad, USA). The image J software was used to calculate the grey value of protein bands.

### Statistical analysis

The GraphPad Prism 9 and SPSS 22.0 software were used for statistical analysis. All experimental data follow a normal distribution and are presented in the form of mean (SD). The comparison between two sets of data was conducted using independent t-test analysis, while one-way analysis was used for multiple sets of data and post-processing analysis was conducted. All p values < 0.05 in the experimental data were considered statistically significant.

## Results

### High expression of HK2 in damaged human knee cartilage and inflammatory stimulation of chondrocytes from mice

**Figure 1:**
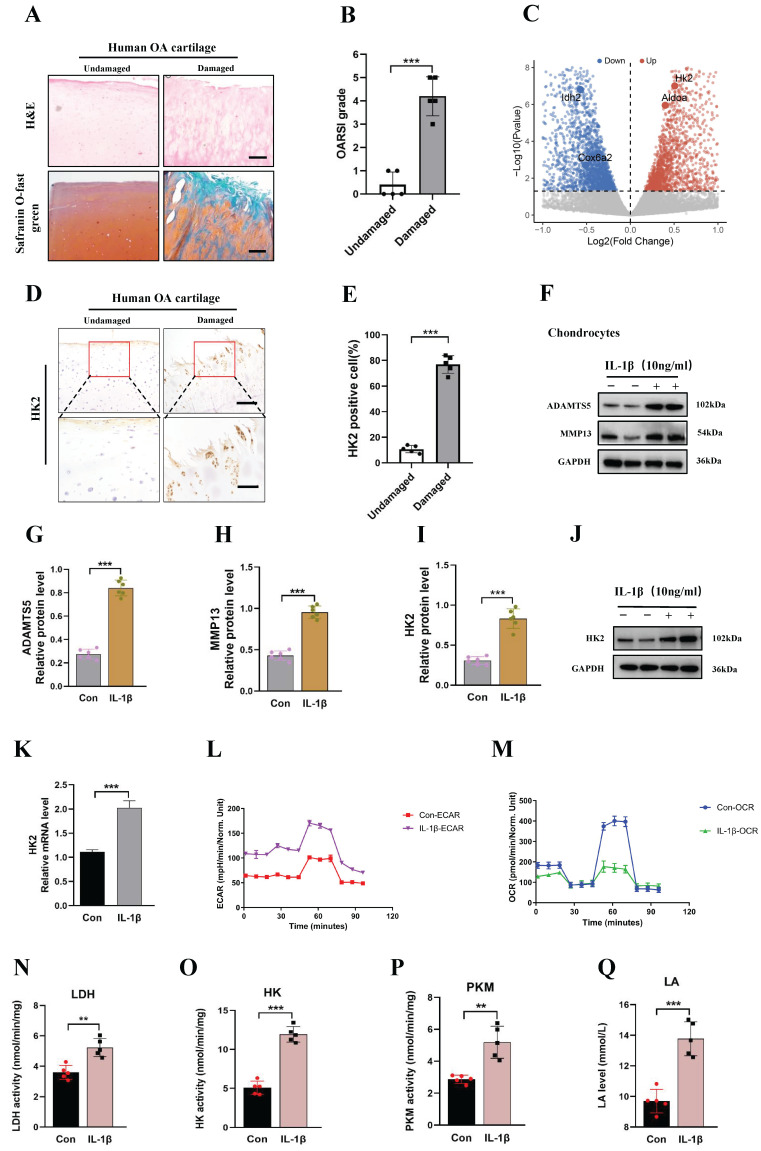
** Damaged human knee cartilage and inflammatory stimulation of chondrocytes from mice express high levels of HK2. (A-B)** Human OA knee joint tissues were harvested and detected using Safranin O-fast green and H&E staining (scale bars, 100 μm), and cartilage damage was evaluated using the Osteoarthritis Research Society International (OARSI) system. ***p < 0.001 (n=5). **(C)** Differential changes in HK2 between chondrocytes from mice in the control group and IL-1β-induced chondrocytes. **(D-E)** Immunohistochemistry (IHC) of HK2 content (scale bars, 100 μm). Graph showing the percentage of HK2-positive cells in undamaged and damaged cartilage tissues. ***p < 0.001 (n=5).** (F-J)** Western blot analysis of ADAMTS5, MMP13, and HK2 protein expression in control chondrocytes and IL-1β-induced chondrocytes, and quantification of WB analysis. ***p < 0.001.** (K)** Relative HK2 mRNA expression from RT-qPCR. ***p < 0.001. **(L-M)** Measurement of the oxygen consumption rate (OCR) and extracellular acidification rate (ECAR) of chondrocytes using a hippocampal cell energy metabolizer (Control group and IL-1β group). (N-Q) Chondrocytes biological activities of lactic dehydrogenase (LDH), pyruvate kinase (PKM), and HK; the lactic acid (LA) levels were tested according to kits (Control group and IL-1β group), (n=5), **p < 0.01, ***p < 0.001. Data are presented as mean (SD).

First, we collected cartilage samples from patients with OA. Using Safranin O-fast green and Hematoxylin and Eosin (H&E) staining, we found that the superficial region of undamaged articular cartilages appeared to be smooth. By contrast, the superficial region of damaged cartilages exhibited cracks and fissures, with abundant surface erosions extending to subchondral bone (Figures 1A and 1B). We analyzed the dataset and found that the HK2 was increased in IL-1β-induced primary chondrocytes from mice (Figure 1C) 30. To further validate the results of the dataset, the genes involved in glycolysis and fermentation, including HK2 expression, were assessed in cartilage samples from patients with OA and IL-1β-induced primary chondrocytes from mice. Chondrocytes in the damaged regions displayed more intense HK2 staining compared with cells in the undamaged areas (Figures 1D and 1E). After treatment of chondrocytes with IL-1β resulted in a significant increase in the expression of catabolic genes (MMP3, MMP13, and ADAMTS5) and inflammatory genes such as IL-6, while decreasing the expression of chondrocyte anabolic factors, such as Aggrecan and COL2A1 (Figures 1F-1H; S1A-S1G). Furthermore, we examined if the expression of HK2 was mediated by IL-1β. We detected a significant increase in mRNA and protein expression of HK2 in IL-1β-induced chondrocytes (Figures 1I-1K). Furthermore, the Immunofluorescence (IF) staining of HK2 in chondrocytes revealed that HK2 expression was increased in IL-1β-induced chondrocytes (Figures S1H and S1I). Further, using seahorse assay, we detected increased glycolysis rate (ECAR) and decreased oxygen consumption rate (OCR) in response to IL-1β as indicated by changes in cellular respiration (Figures 1L and 1M). Finally, we confirmed the increased glycolysis by detecting elevated levels of intracellular HK, lactic dehydrogenase (LDH), pyruvate kinase (PKM) activity, and extracellular lactic acid (LA) in the IL-1β-treated cell cultures (Figures 1N-1Q). Overall, these results suggest that elevated HK2 expression may be involved in the response of chondrocytes from mice to OA-induced conditions.

### Increased HK2 activity and expression in the DMM mice model

**Figure 2:**
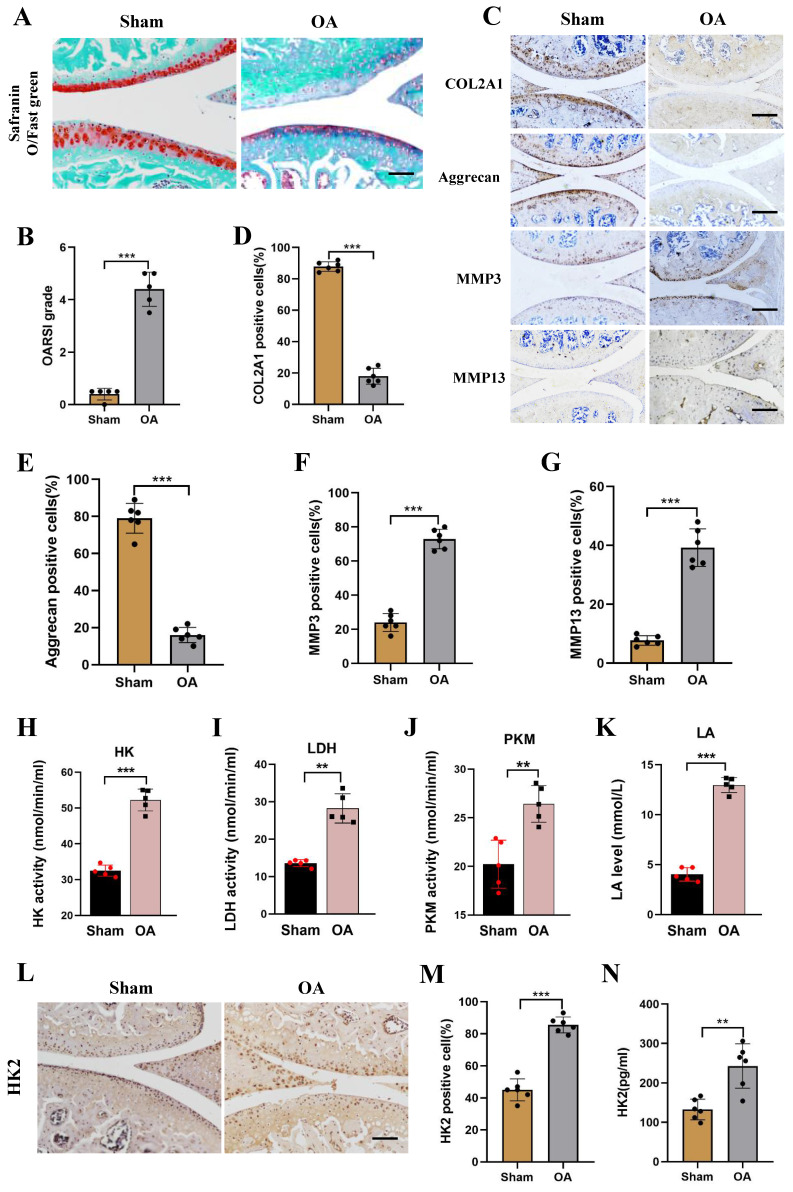
** Increased HK2 activity and expression in DMM mice model. (A-B)** Knee joint tissues of the Sham and OA groups were harvested and detected using Safranin O-fast green staining (scale bars, 100 μm), and cartilage damage was evaluated using the OARIS score. ***p < 0.001, (n=5).** (C-G)** IHC of COL2A1, Aggrecan, MMP3, and MMP13 content (scale bars, 100 μm). Graph showing the percentage of positive cells for COL2A1, Aggrecan, MMP3, and MMP13 in the Sham and OA groups. ***p < 0.001. **(H-K)** Biological activities of LDH, PKM, and HK in chondrocytes as well as LA levels (Sham group and OA group). **p < 0.01, ***p < 0.001.** (L-M)** IHC of HK2 expression (scale bars, 100 μm), Graph showing the percentage of cells positive for HK2 in the Sham group and OA group. ***p < 0.001. **(N)** ELASA assay shows the HK2 expression levels in the OA group and the Sham group, **p < 0.01. Data are presented as mean (SD).

Four weeks after the establishment of the mouse DMM model, the OA phenotype was compared between OA mice and Sham mice. As shown in (Figure S2A), severe subchondral osteosclerosis and increased osteophyte formation were found in the knee joints of OA mice using micro-CT assay. Using Mimics Innovation Suite 21 software for three-dimensional imaging reconstruction of the knee joint, the volume of bone trabeculae in the OA mice was significantly lower than in Sham mice. The analysis revealed that the BV/TV ratio in the OA group was significantly lower than in the Sham group (Figures S2B and S2C). Furthermore, Safranin O-fast green, H&E staining, and Toluidine blue staining were performed and revealed exacerbated damage of OA joint cartilages (Figures 2A and 2B; Figure S2D). Additionally, immunohistochemical staining (IHC) showed down-regulated expression of Aggrecan and COL2A1, while the expression levels of MMP3 and MMP13 were increased in the OA group (Figures 2C-2G). To further confirm the change of glucose metabolism of OA mice, we assessed the activity of glycolysis-related kinase, and observed increased levels of HK, PKM, and LDH activity, and increased LA level (Figures 2H-2K). Furthermore, IHC and ELISA assays showed an increase in HK2 expression level in the OA group compared with the Sham group (Figures 2L-2N). Overall, these results suggest the presence of high levels of expression of HK2 in the joint cartilage of mice with OA.

### HK2 inhibition reduces catabolic activity and increases synthetic metabolism in mouse chondrocytes

**Figure 3:**
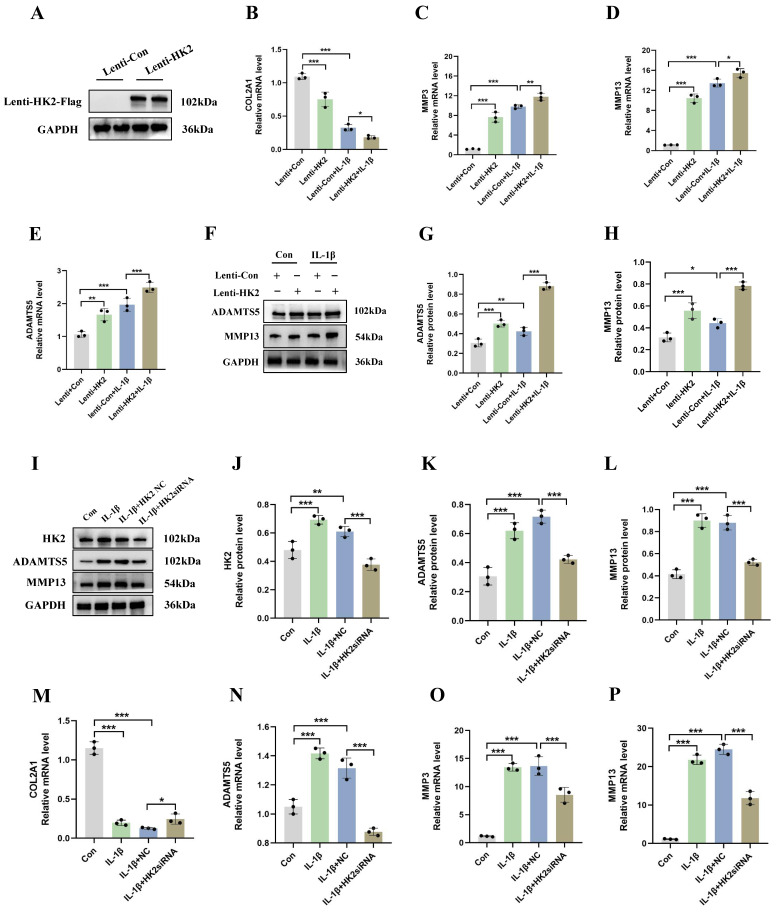
** HK2 inhibition reduces catabolic activity and increases synthetic metabolism in mouse chondrocytes. (A)** Western blot shows that HK2 is successfully overexpressed. **(B-E)** qRT-PCR assay of COL2A1, MMP3, MMP13, and ADAMTS5 mRNA in chondrocytes of each group. *p < 0.05, **p < 0.01, ***p < 0.001. **(F-H)** After overexpression of HK2 in OA chondrocytes, Western blot analysis of ADAMTS5 and MMP13 protein expression in each group and quantification of WB analysis. **p < 0.01, ***p < 0.001. **(I-L)** After suppression of HK2 in OA chondrocytes, Western blot analysis of ADAMTS5 and MMP13 protein expression in each group and quantification of WB analysis. **p < 0.01, ***p < 0.001. **(M-P)** qRT-PCR assay of COL2A1, MMP3, MMP13, and ADAMTS5 mRNA in chondrocytes of each group after suppression of HK2 in OA chondrocytes, ***p < 0.001, Data are presented as mean (SD).

To determine the role of HK2 in the pathogenesis of OA, we transduced a mouse chondrocyte cell line (ATDC5) with lentiviral particles overexpressing HK2 and investigated its effects on both normal (non-IL-1β-induced) and IL-1β-induced cells. Our results indicated that in normal cells (Lenti+Con and Lenti+HK2 without IL-1β treatment), HK2 overexpression significantly increased the mRNA levels of catabolic molecules, including ADAMTS5, MMP3, and MMP13, while decreasing the mRNA levels of COL2A1. In IL-1β-induced chondrocytes, HK2 overexpression further elevated the mRNA levels of these catabolic molecules and suppressed COL2A1 expression (Figures 3A-3E). Additionally, Western blot analysis confirmed that in both normal and IL-1β-induced chondrocytes, HK2 overexpression led to increased levels of ADAMTS5 and MMP13 (Figures 3F-3H). These findings suggest that HK2 overexpression promotes ECM degradation in chondrocytes under both normal and inflammatory conditions, contributing to the pathogenesis of OA. Furthermore, we transfected chondrocytes with HK2siRNA, the HK2-NC as a control, treated the chondrocytes with IL-1β, and then assessed the expression levels of key molecules associated with OA phenotypes. Our results indicated that HK2siRNA significantly reduced the expression levels of HK2, decreased the IL-1β-induced increase protein levels of molecules related to ECM degradation when compared with the control group (Figures 3I-3L). Furthermore, the suppression of the HK2 decreased the expression of ADAMTS5, MMP3, and MMP13. Conversely, the suppression of HK2 increased the expression levels of COL2A1 (Figures 3M-3P).

### HK2 promotes catabolism in OA chondrocyte by inhibiting HMGA2

**Figure 4:**
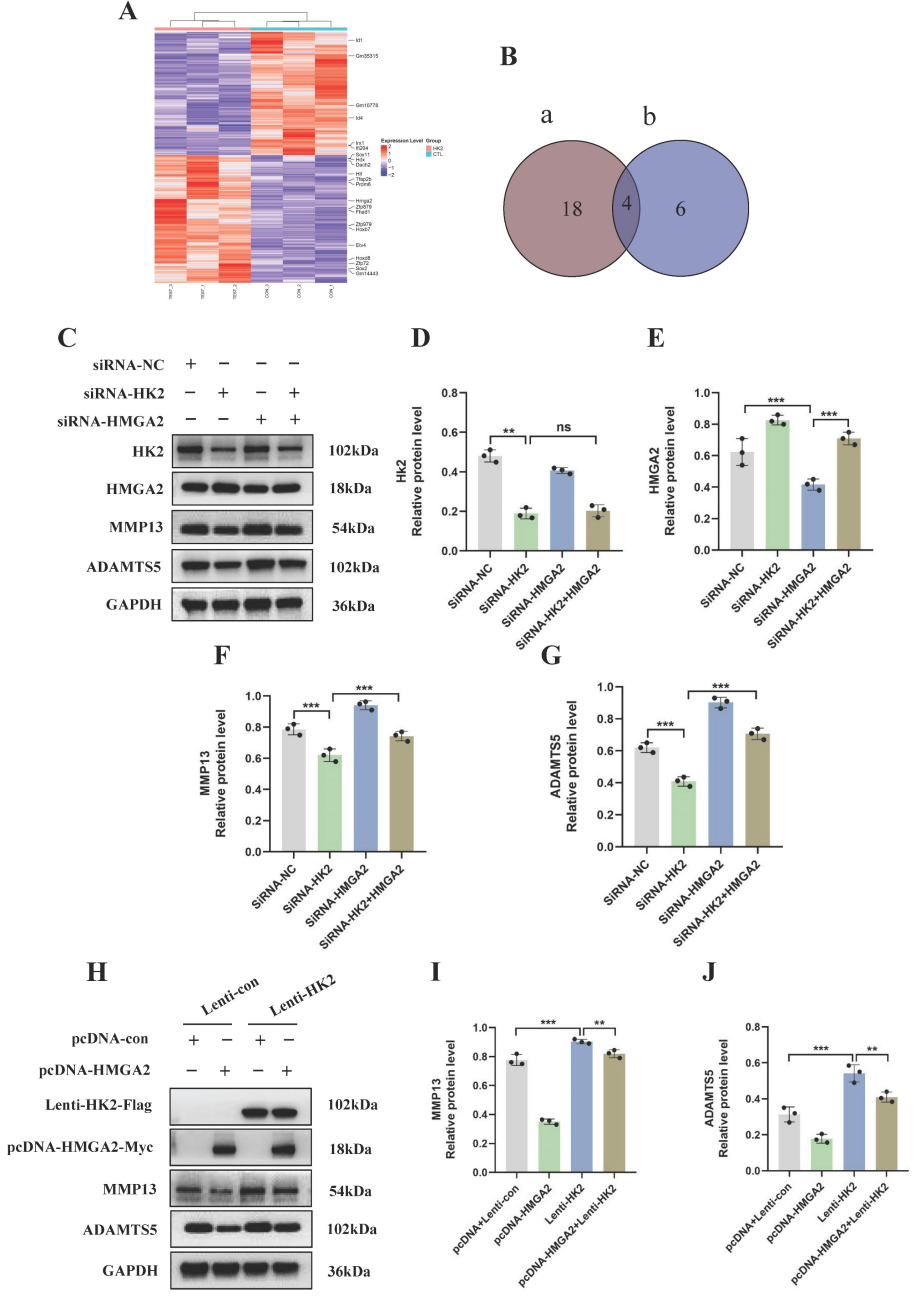
** HK2 promotes catabolism in OA chondrocytes by inhibiting HMGA2. (A)** DEseq2 analysis revealed differentially expressed genes and transcriptional regulatory factors. **(B)** Venn showing candidate genes among the intersecting genes. **(C-G)** Knocked down HK2 and HMGA2 in IL-1β-induced OA chondrocytes to assess the MMP13 and ADAMTS5 expression using Western blot assay and quantification of WB analysis. **p < 0.01, ***p < 0.001. **(H-J)** Overexpressed HK2 and HMGA2 in IL-1β-induced OA chondrocytes to assess the MMP13 and ADAMTS5 expression using Western blot assay and quantification of WB analysis, **p < 0.01, ***p < 0.001. Data are presented as mean (SD).

To further elucidate the molecular mechanisms underlying the occurrence and development of OA and HK2, we conducted transcriptome sequencing after knocking down HK2 in the OA chondrocytes from mice. DEseq2 analysis and pathway enrichment revealed that 142 genes were upregulated and 102 genes were downregulated following HK2 knockdown (Figure 4A; Figure S3A). We selected 22 transcription factors with significant expression differences and screened 10 transcription factors closely related to the disease progression of OA through a literature review (Figure 4B). Ultimately, we chose HMGA2 as a candidate gene among the intersecting genes. We hypothesized that IL-1β-induced HK2 targeting regulated HMGA2, thereby promoting the catabolic capacity in OA. HMGA2 plays a crucial role in inducing oxidative phosphorylation (OXPHOS) to metabolic reprogramming of glycolysis Additionally, HMGA2 is also a significant factor in the metabolic reprogramming of cartilage in OA. Upon knocking down HK2 in IL-1β-induced chondrocytes, the results revealed a significant decrease in both the mRNA and protein levels of HMGA2 in the OA chondrocytes (Figures S3B-S3E). To clarify whether HK2 promoted OA-induced cartilage damage caused by inhibiting HMGA2, we concurrently knocked down HK2 and HMGA2 in IL-1β-induced OA chondrocytes to assess the expression levels of proteins associated with OA cartilage degradation. Western blot results indicated that knocking down HK2 inhibited the expression levels of ADAMTS5 and MMP13 proteins compared to the control group (Figures 4C-4G). However, simultaneous suppression of HK2 and HMGA2 led to a significant increase in the expression levels of ADAMTS5 and MMP13 compared to the siRNA-HK2 group (Figures 4C-4G). These findings suggested that suppression of HMGA2 counteracted the changes in catabolic activity in OA induced by HK2 knockdown.

Our results indicate that inhibiting HMGA2 in chondrocytes can decrease the therapeutic impact against OA that is mediated by HK2 knockdown. Afterwards, in stably transfected cells of overexpressing HK2, we examined the expression levels of OA cartilage degradation-related proteins. The results of Flag and Myc tags indicated successful overexpression of both HK2 and HMGA2. Compared to the Lenti-HK2 group, simultaneous overexpression of HK2 and HMGA2 led to a significant decrease in the protein levels of ADAMTS5 and MMP13 (Figures 4H-4J). These results suggest that the overexpression of HMGA2 partially reversed the OA cartilage damage induced by overexpressing HK2 in chondrocytes.

### Impact of PEMF on the synthesis, catabolism levels, and glycolytic activity of OA chondrocytes

**Figure 5:**
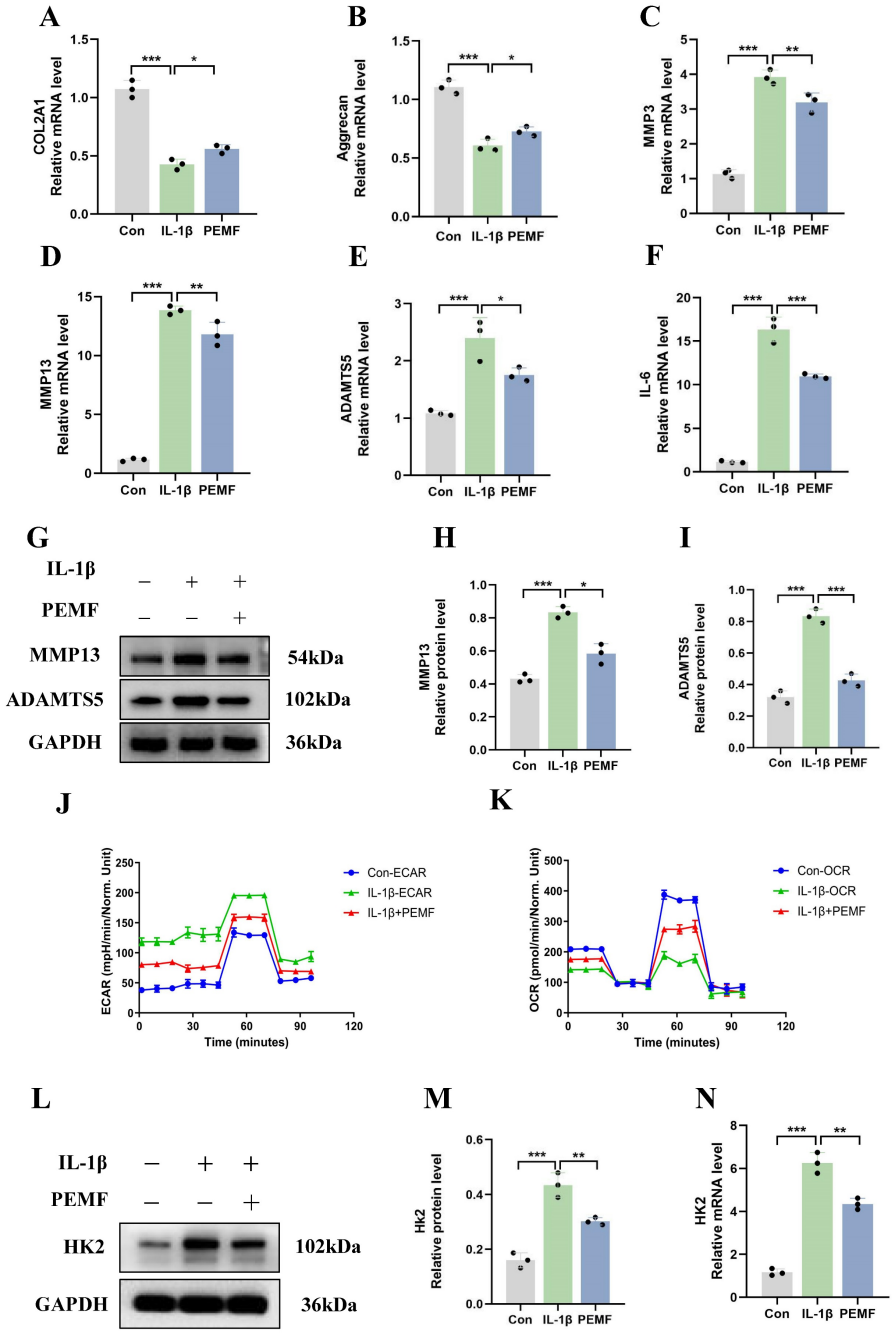
** The impact of PEMF on the synthesis, catabolism levels, and glycolysis activity of OA chondrocytes. (A-F)** After PEMF intervention in IL-1β-induced mouse chondrocytes, qRT-PCR analysis of COL2A1, Aggrecan, IL-6, ADAMTS5, MMP3, and MMP13 in OA chondrocytes in each group. *p < 0.05, **p < 0.01, ***p < 0.001. **(G-I)** After PEMF intervention in IL-1β-induced mouse chondrocytes, Western blot analysis of ADAMTS5 and MMP13 protein expression in each group, and quantification of WB analysis. *p < 0.05, ***p < 0.001. **(J-K)** After PEMF intervention in IL-1β-induced mouse chondrocytes, the hippocampal cell energy metabolizer was used to measure the OCR and ECAR of chondrocytes.** (L-N)** After PEMF intervention in IL-1β-induced mouse chondrocytes, HK2 mRNA and protein levels were assessed using Western blot assay and qRT-PCR analysis, **p < 0.01, ***p < 0.001. Data are presented as mean (SD).

This study used PEMF to intervene IL-1β-induced mouse chondrocytes. The qRT-PCR results revealed that, compared to the OA group, the mRNA expression levels of COL2A1 and Aggrecan increased after PEMF intervention (Figures 5A and 5B). Conversely, PEMF intervention suppressed the mRNA expression levels of IL-6, ADAMTS5, MMP3, and MMP13 in OA chondrocytes (Figures 5C-5F). Western blot analysis of MMP13 and ADAMTS5 expression in OA chondrocytes also provided results that were consistent with the above findings (Figures 5G-5I). These findings suggested that PEMF could significantly inhibit synthesis and catabolism, as well as the inflammatory response in OA chondrocytes.

We aimed to investigate whether PEMF can decrease glycolytic activity in OA chondrocytes. Therefore, we examined changes in glycolysis rate and oxygen consumption rate in chondrocytes. Compared to the IL-1β group, the PEMF intervention significantly reduced ECAR and increased the OCR (Figures 5J and 5K). Furthermore, PEMF intervention in OA chondrocytes led to a notable reduction in the activities of HK, PKM, and LDH, along with a significant decrease in LA levels (Figures S4A-S4D). Overall, these data provided evidence that PEMF can influence the metabolic shift in OA chondrocytes, thereby reducing glycolytic rates and glycolytic activity. Furthermore, after using PEMF on OA chondrocytes, we assessed the mRNA and protein levels of HK2 and HMGA2. The qRT-PCR results revealed that, compared to the IL-1β group, there was a significant decrease in HK2 mRNA expression levels and an increase in HMGA2 expression levels after PEMF intervention (Figures 5L-5N; Figures S4E-S4G). These findings were further confirmed by Western blot analysis. Therefore, we confirmed that PEMF intervention can suppress the expression of HK2 in OA chondrocytes while promoting the expression of HMGA2, thereby mitigating the effects of OA.

### PEMF decreases the level of catabolism in OA through the HK2/HMGA2 signaling pathway

**Figure 6:**
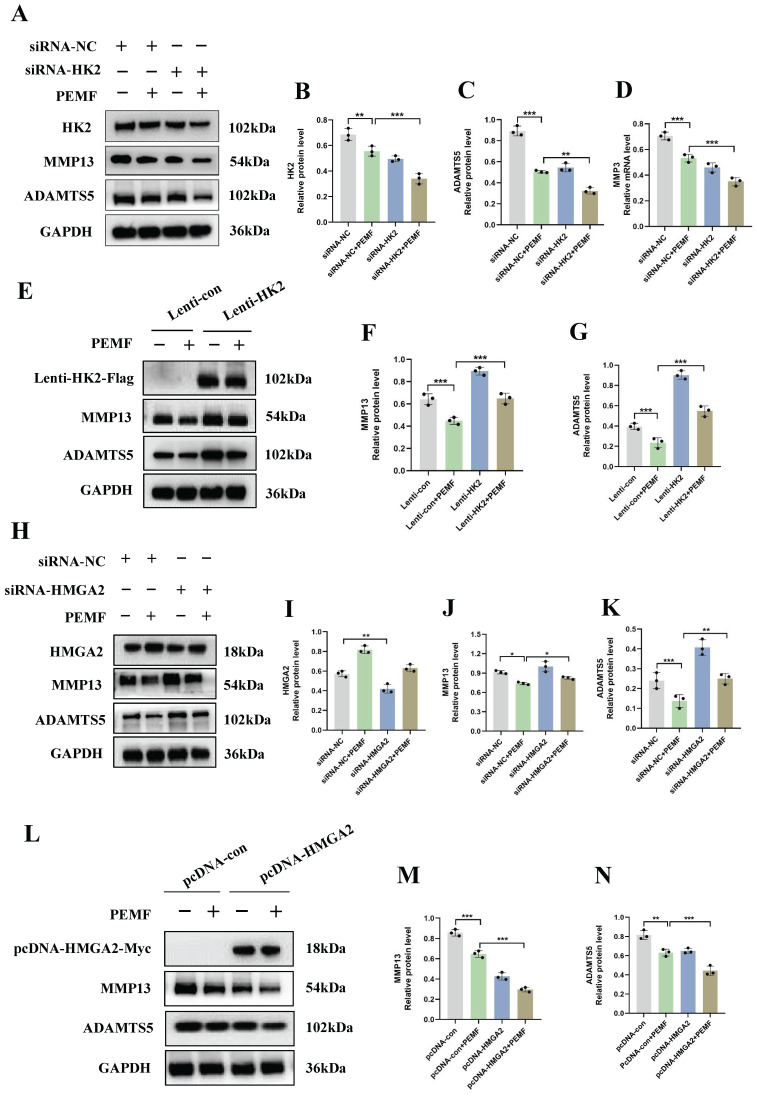
** PEMF decreases the catabolism levels in OA through the HK2/HMGA2 signaling pathway. (A-D)** Knocked-down HK2 while applying PEMF intervention; the protein expression levels of HK2, MMP13, and ADAMTS5 in OA chondrocytes were detected using Western blot and quantification of WB analysis. ***p < 0.001. **(E-G)** Overexpressed HK2 while applying PEMF intervention; the protein expression levels of MMP13 and ADAMTS5 in OA chondrocytes were detected using Western blot and quantification of WB analysis was performed. ***p < 0.001.** (H-K)** Knocked-down HMGA2 while applying PEMF intervention; the protein expression levels of HK2, MMP13, and ADAMTS5 in OA chondrocytes were detected using Western blot and quantification of WB analysis. *p < 0.05, **p < 0.01, ***p < 0.001. **(L-N)** Overexpressed HMGA2 while applying PEMF intervention; the protein expression levels of MMP13 and ADAMTS5 in OA chondrocytes were detected using Western blot and quantification of WB analysis, *p < 0.001, ***p < 0.001. Data are presented as mean (SD).

To elucidate the mechanism underlying the effects of PEMF on cartilage homeostasis, OA chondrocytes were transduced with HK2 overexpression lentiviral particles, HMGA2 overexpression plasmid, HK2siRNA, and HMGA2siRNA. Our results demonstrated that, compared to the siRNA-NC+PEMF group, knocked-down HK2 after PEMF intervention was associated with a significant decrease in the protein expression levels of HK2, MMP13, and ADAMTS5 in OA chondrocytes (Figures 6A-6D). Furthermore, interfering with HMGA2 can counteract the PEMF-induced increase in protein levels of HMGA2, and decrease the protein levels of ADAMTS5 and MMP13 (Figures 6H-6K).

Furthermore, we investigated whether the overexpression of HK2 and HMGA2 individually affected the therapeutic effect of PEMF on OA. The findings of this study indicated that, compared to the Lenti-con+PEMF group, the protein levels of ADAMTS5 and MMP13 significantly increased after using PEMF intervention in chondrocytes with HK2 overexpression (Figures 6E-6G). Conversely, compared to chondrocytes overexpressing HMGA2 alone, simultaneous overexpression of HMGA2 with PEMF intervention in chondrocytes resulted in a significant decrease in the protein levels of MMP13 and ADAMTS5 (Figures 6L-6N). In summary, our results clearly indicated that PEMF decreased the level of catabolism in OA cartilages through the HK2/HMGA2 signaling pathway.

### Inhibiting HK2 promotes PEMF to alleviate joint cartilage damage in OA

We previously validated that in OA chondrocytes, targeted inhibition of HK2 expression can enhance the ability of PEMF to alleviate the balance between cartilage degradation and synthesis metabolism, thereby mitigating the effects of OA. We further demonstrated *in vivo* whether inhibiting HK2 expression could promote the PEMF-mediated reduction in osteophyte formation and cartilage degeneration caused by OA. We established an *in vivo* mouse OA model by using adeno-associated virus (AAV) to interfere with HK2 expression. One month after DMM surgery, AAV-HK2shRNA was injected into the joint cavity (using AAV-GFP as controls). Initially, we examined the impact of AAV-HK2shRNA on the articular cartilage. Cartilage samples were then isolate, and micro-CT was performed. As shown in Figures S5A and S5B, osteophyte formation was observed in the OA+GFP-AAV group, which was largely abolished in the OA+AAV- HK2shRNA group. Furthermore, significantly increased BV/TV were observed in the OA+AAV-HK2shRNA group (Figure S5C). The HE staining results demonstrated that the OA+AAV-HK2shRNA group retained the overall integrity of the articular cartilage compared to the OA+AAV-GFP group (Figures S5D).

**Figure 7:**
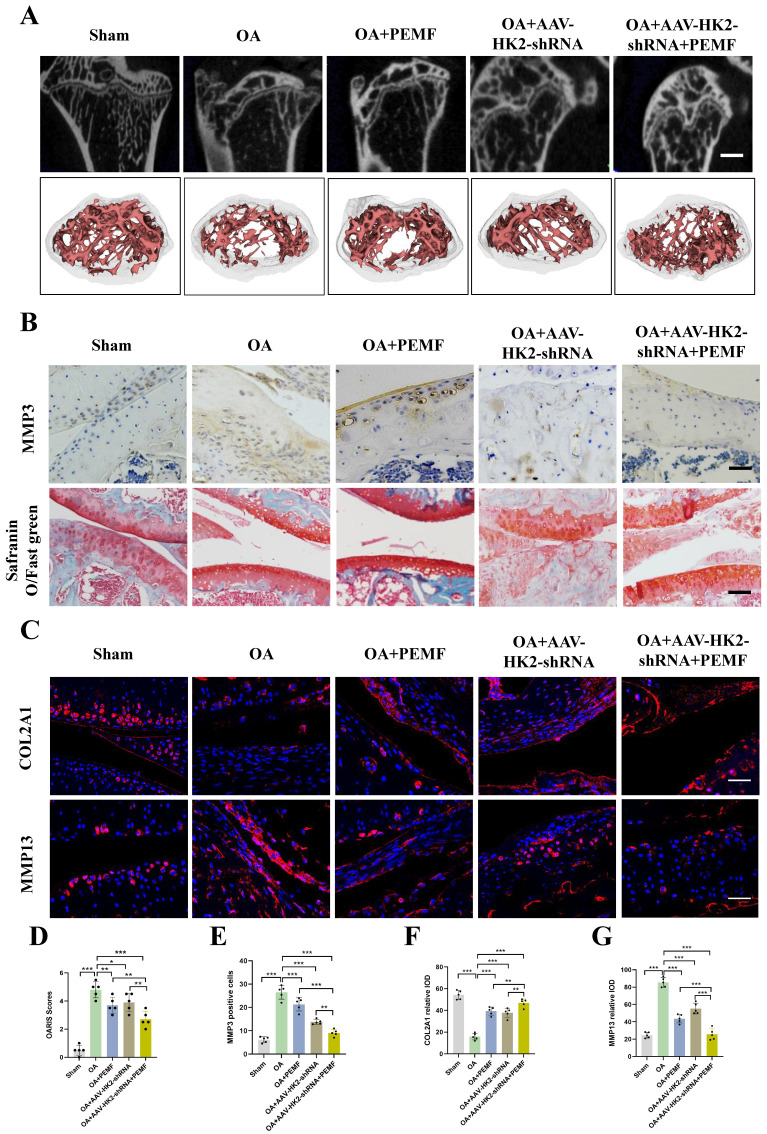
** Inhibiting HK2 promotes PEMF to alleviate joint cartilage damage in mice caused by OA. (A)** Cartilage samples were isolated and micro-CT was performed in each group, and the three-dimensional imaging reconstruction of the knee joint using Mimics Innovation Suite 21 software (n=5). **(B)** IHC of MMP3 expression (scale bars, 100 μm) in mice OA knee joint tissues detected using Safranin O-fast green staining (scale bars, 100 μm). **(C)** IF of COL2A1 and MMP13 expression (scale bars, 100 μm). **(D)** Cartilage damage evaluated using the Osteoarthritis Research Society International (OARSI) system. *p < 0.05, **p < 0.01, ***p < 0.001. **(E)** Graph showing the percentage of positive expression of MMP3, COL2A1, and MMP13 in each group, **p < 0.01, ***p < 0.001. Data are presented as mean (SD).

Furthermore, using an adeno-associated virus (AAV)-based *in vivo* mouse OA model, we investigated whether AAV-HK2 interference could enhance the PEMF-mediated regulation of the balance between catabolism and anabolism, and mitigate cartilage damage in OA. Following the completion of PEMF intervention, we assessed cartilage degeneration through micro-CT and histological examinations. The results indicated a more pronounced decrease in osteophyte formation in the OA+AAV-HK2shRNA, OA+PEMF, and OA+AAV-HK2shRNA+PEMF groups compared to the OA group (Figure 7A; Figure S5E). Particularly, the OA+AAV-HK2shRNA+PEMF group exhibited milder osteophyte formation compared to the OA+PEMF group; this phenomenon was confirmed by the results of BV/TV. The H&E and Safranin O-fast green staining results indicated that compared to the OA group, the integrity of the articular cartilage was better preserved in the OA+AAV-HK2shRNA group, OA+PEMF group, and OA+AAV-HK2shRNA+PEMF group (Figure 7B). The OARIS score results showed that, compared to the OA+PEMF group, the OA+AAV-HK2shRNA+PEMF group had milder cartilage damage (Figure 7D). Additionally, we investigated the effect of AAV-HK2shRNA and PEMF on the expression of proteins involved in catabolism and anabolism. Our results showed that, compared to the OA group and OA+PEMF intervention group, the expression of COL2A1 significantly increased, while the expression of catabolism genes MMP3 and MMP13 significantly decreased in the OA+HK2-shRNA+PEMF group (Figures 7B and 7C, Figures 7E-7G). Finally, we used reagent kits to detect the activity of glycolytic kinases and the LA level in mouse serum. The results showed that the activity of HK, PKM, and LDH in the OA+PEMF group was significantly lower than that in the OA+AAV-HK2shRNA+PEMF group, and the glycolytic activity in the OA+AAV-HK2shRNA+PEMF group was significantly lower than that in the OA+PEMF intervention group (Figures S5F-S5I). The above results indicated that inhibiting HK2 could promote PEMF to alleviate osteophyte formation and reduce catabolic metabolism levels and glycolytic activity, thereby reducing OA progression.

### HK2 inhibitor combined with PEMF decrease OA cartilage damage in mice

**Figure 8:**
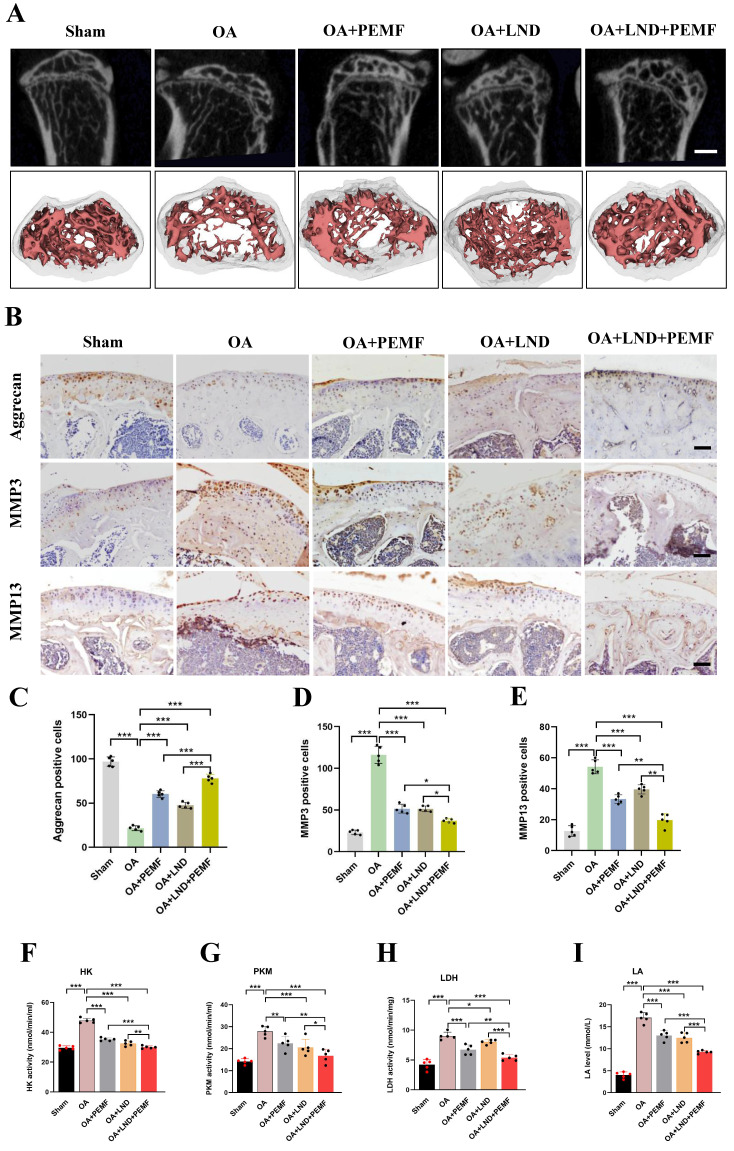
** HK2 inhibitor combined with PEMF better decreases cartilage damage in mouse OA model.** (A) After PEMF intervention and HK2 inhibitor, Lonidamine (LND), injection, the cartilage samples in each group were isolated and examined using micro-CT, and three-dimensional imaging reconstruction of the knee joint was done using Mimics Innovation Suite 21 software (n=5). (B) IHC of aggrecan, MMP3, and MMP13 expression (scale bars, 100 μm). (C) Graph showing the percentage of positive expression of aggrecan, MMP3, and MMP13 in each group. **p < 0.01, ***p < 0.001. (D-G) After PEMF intervention and LND injection, the mice biological activities of LDH, PKM, and HK, the LA levels were tested according to the kits, (n=5), *p < 0.05, **p < 0.01, ***p < 0.001. The data are presented as mean (SD).

Our results showed that in OA chondrocytes, reducing HK2 expression can enhance PEMFs therapeutic effect against OA. Therefore, *in vivo* experiments were conducted to further validate this result. The HK2 inhibitor Lonidamine (LND) was used to suppress HK2 expression. Mice joint samples were collected, and micro-CT was performed. As shown in Figure 8A and Figure S6A, osteophyte formation in the knee joint of the DMM model was alleviated using the PEMF intervention, monotherapy with LND, combined treatment of PEMF and LND. Of note, PEMF and LND combined treatment was most effective in preventing osteophyte formation, indicating an enhanced effect of the combination. HE staining revealed that, after DMM surgery, there was evident damage to the articular cartilage surfaces of the femoral condyles and tibias. Monotherapy with LND or PEMF intervention slightly alleviated cartilage damage; however, the extent of damage remained higher than in the group treated with PEMF and LND combined treatment (Figure S6A). Additionally, Safranin O-fast green staining and OARSI results indicated that monotherapy with LND, PEMF intervention, and PEMF and LND combined treatment all led to lower OARSI scores compared to scores in the OA group; however, PEMF and LND combined treatment exhibited lower OARSI scores compared to the other groups (Figures S6B and S6C).

We also used IHC to detect the localization and expression of aggrecan, MMP3, and MMP13. Compared to the expression in the OA group, the expression levels of aggrecan in the knee joint of the DMM model was higher after PEMF intervention, monotherapy with LND, and PEMF and LND combined treatment; however, PEMF and LND combined treatments were most effective in increasing Aggrecan expression. In contrast, PEMF and LND combined treatment was most effective in decreasing MMP3 and MMP13 expression, compared with those of PEMF intervention and monotherapy with LND. This observation suggested that PEMF and LND combined treatment mitigated DMM-induced increase in catabolic levels (Figures 8B-8E). Based on the significant increase in glycolytic activity observed in the serum of OA mice, we further investigated the combined effects of PEMF and LND treatment on glycolytic activity in OA mice. The results showed that monotherapy with LND, PEMF intervention, and PEMF and LND combined treatment all led to reduced serum activities of HK, PKM, and LDH, along with a decrease in the LA level. Furthermore, compared to the group that received only PEMF intervention or monotherapy with LND, the group that received PEMF and LND combined therapy exhibited significantly lower serum activities of HK, PKM, and LDH, as well as a more pronounced reduction in LA levels (Figures 8F-8I). Hence, our results suggest that the combination of HK2 inhibitor LND and PEMF could better reduce glycolytic activity, osteophyte formation, and cartilage degeneration in OA mice.

## Discussion

As the population ages, OA being a chronic degenerative joint disease is receiving increasing attention 31. Epidemiological surveys indicate that the prevalence of OA in populations aged 50 years and above in developed countries is as high as 60%, and medical expenses for treating OA account for 1.0-2.5% of the gross domestic product in some countries, imposing a significant economic burden on families and society 32, 33.

The clinical manifestations of OA primarily include pain, deformity, and joint dysfunction, leading to the development of additional psychological disorders, such as depression and anxiety. Systematic reviews and meta-analyses suggest a clear positive correlation between the severity of OA pain and anxiety and depression 32, further emphasizing the urgency of addressing OA. OA is a complex and heterogeneous musculoskeletal disease that can affect multiple joints, such as the knee, lumbar spine, and temporomandibular joint 34, 35. Effective methods for cartilage repair are still lacking; however, a better understanding of the pathogenesis of OA may lead to the identification of new therapeutic targets and approaches 36.

Recent research suggests that the abnormal metabolism of chondrocytes represents stress-induced alterations in the OA inflammatory microenvironment, potentially playing a crucial role in the process of cartilage destruction in OA 9. Under external stimuli (e.g., mechanical loading, insulin resistance, and stress) chondrocytes undergo metabolic pathway transitions to adapt to changes in the microenvironment for survival, including shifts from OXPHOS to glycolysis 9. Therefore, determining the metabolic pathways of chondrocytes is crucial for unraveling the pathogenesis of OA. Glycolysis represents a major process for breaking down monosaccharides, involving various physiological and pathological activities, such as energy metabolism, maintenance of microenvironment homeostasis, musculoskeletal functions, and neural transmission 37. Among them, HK2, LDHA, and PKM are key rate-limiting enzymes in the glycolysis 38. Our research results indicate that in OA chondrocytes, the activity of key glycolytic enzymes (HK, PKM, and LDH) is increased, the LA level and ECAR are also increased, while OCR is decreased. Furthermore, using *in vivo* experiments, we identified increased activities of HK, PKM, LDH, and LA levels in the serum of OA mice compared to control mice. Therefore, our findings suggest the occurrence of a metabolic shift from OXPHOS to aerobic glycolysis in OA chondrocytes, leading to an increase in glycolytic rate and glycolytic activity. HK2 is the first catalytic enzyme in the glycolytic process and also functions as a glucose sensor 15, which can convert glucose to G-6-P 39. Studies have indicated that HK2 is highly expressed in fibroblast-like synovial cells (FLS) of rheumatoid arthritis (RA) and OA, and hypoxia and TNF induction can increase the expression of HK2 in FLS 16. Our study revealed increased expression of HK2 in the cartilage tissues of patients with OA, the articular cartilage of OA mice, and OA chondrocytes. This study demonstrated that knocking down HK2 in OA chondrocytes could inhibit catabolism and inflammatory responses and promote anabolic processes. Conversely, overexpression of HK2 significantly increased OA catabolism and inflammatory responses while inhibiting the levels of cellular synthesis. Therefore, our findings suggest that HK2 may be an effective therapeutic target for OA, as HK2 disrupts the balance between anabolism and catabolism, promotes inflammatory responses, and thus plays a crucial role in the occurrence and development of OA.

The molecular mechanisms by which HK2 participates in the progression of OA are currently unclear. Therefore, in this study, we knocked down HK2 in an OA chondrocyte model and performed transcriptome sequencing. We identified the differentially regulated transcription factor HMGA2, and confirmed through qRT-PCR and Western blotting that knocking down HK2 significantly upregulated the mRNA and protein expression levels of HMGA2. Moreover, HMGA2, a member of the HMGA family, regulates nuclear chromatin structure. It targets key signaling pathways and plays a crucial role in processes such as cell apoptosis, proliferation, and invasion 40. Studies have indicated that the HMGA2 protein can bind to the D-loop region of mitochondrial DNA (mtDNA), and the endoplasmic reticulum stress/HMGA2 axis mediates metabolic reprogramming from OXPHOS to aerobic glycolysis 41. Other studies have indicated that HMGA2 can mediate cell metabolism reprogramming by influencing autophagic activity 42, hence it plays an important role in inducing metabolic reprogramming from OXPHOS to glycolysis 42. In summary, our findings indicate that HMGA2 plays a significant role in aerobic glycolysis. A previous study indicated that activation of the HMGA protein family can modulate the balance between chondrocyte anabolism and catabolism, thereby reversing OA cartilage damage 43. Another report suggests that HMGA2 activates chondrocyte reprogramming by binding to the SOX9 promoter regulatory region, further highlighting the mediation of the Let-7 effect by HMGA2, whose activation results in chondrocyte anabolism and catabolism, thereby reversing the OA features 19. Accordingly, in this study, we simultaneously inhibited or overexpressed both HK2 and HMGA2 in OA chondrocytes. Our findings suggest that suppressing HMGA2 expression could inhibit the decrease in OA catabolism levels mediated by interfering with HK2. Conversely, overexpressing HMGA2 can partially reverse the increase in OA catabolism levels mediated by overexpressing HK2. Overall, these findings confirm the significant role of the HK2/HMGA2 signaling axis in OA cartilage damage, providing ample evidence for future targeted HK2 therapy in OA.

As a non-invasive physical therapy, PEMF has gained wide recognition in the prevention and treatment of various diseases. Studies have reported that PEMF can exert protective effects on various diseases by regulating oxidative stress 24, 25, mitochondrial dysfunction, and energy metabolism 26. Glycolysis is a crucial aspect of energy metabolism; however, its relationship with PEMF has not been extensively studied. Our experimental results demonstrated that PEMF could inhibit the expression of HK2 and reduce glycolytic activity and glycolysis rate in OA chondrocytes. Additionally, this study found that PEMF decreases the catabolism levels of chondrocytes, which was significantly inhibited after overexpressing HK2. Conversely, when HK2 expression was suppressed, the ability of PEMF-reduced catabolism levels was enhanced in chondrocytes. *In vivo*, we inhibited HK2 expression in mice knee joints using AAV-HK2shRNA and conducted PEMF intervention. Our results showed that inhibiting HK2 could promote PEMF to alleviate osteophyte formation and reduce OA joint cartilage damage. This study validated whether PEMF alters the expression levels of HMGA2, a key downstream signaling molecule of HK2. Our results indicated that PEMF upregulated the mRNA and protein expression levels of HMGA2. Further interference and overexpression of HMGA2 revealed that the downregulation of HMGA2 in the OA chondrocytes counteracted the beneficial effects of PEMF on cartilage damage, while the overexpression of HMGA2 decreased the expression of degradation metabolism genes, thereby promoting the beneficial effects of PEMF on cartilage damage. The above results suggest that PEMF ameliorates cartilage damage caused by OA by inhibiting HK2 expression and upregulating HMGA2 expression.

The combined use of multiple treatment modalities is a common clinical approach for treating diseases. In this study, the inhibitory effects of both HK2 suppression and PEMF on glycolytic activity, as well as the imbalance between anabolism and catabolism levels induced by OA, were validated *in vitro*. Subsequently, the study investigated the therapeutic efficacy of the HK2 inhibitor, LND, in combination with PEMF intervention using *in vivo* experiments. LND, a derivative of indole-3-carboxylic acid, was initially reported as a contraceptive and later repurposed as an anti-tumor drug due to its anti-Warburg effect and inhibition of HK2 activity 44. Furthermore, studies demonstrated that injection of LND in animal models of ischemic brain injury 44 and OA 45 significantly reduced HK2 expression levels and exhibited anti-inflammatory effects. Additionally, under lipopolysaccharide and hypoxic stimulation, LND application successfully inhibited HK2, leading to a reduction in the release of inflammatory factors in macrophages and microglial cells, thereby alleviating the severity of arthritis, further highlighting its potential for treating inflammatory diseases 46. Another study also suggested that LND could inhibit HK2 activity and reduce TGF-β-induced fibrosis through its pharmacological effects 47. Overall, LND can prevent and treat diseases by inhibiting HK2 activity and expression levels; hence, it is a suitable candidate drug for the clinical treatment of inflammatory diseases. This study employed the combination of LND and PEMF to treat OA. The results demonstrated that both individual interventions, monotherapy with PEMF or monotherapy with LND, as well as a combination of the two methods, could ameliorate cartilage degeneration, osteophyte formation, glycolytic activity, and restore the balance between extracellular matrix degradation and synthesis induced by OA in mice. However, compared to monotherapy using PEMF, the simultaneous administration of LND and PEMF was more effective in improving the OA phenotype. Therefore, the findings of this study suggest that the combination of the HK2 inhibitor, LND, with PEMF significantly alleviates cartilage degeneration and inhibits subchondral bone reconstruction in OA, offering a novel strategy for OA treatment.

This study highlighted several findings that have important implications for understanding the role of HK2 in OA progression. We made the following conclusions: 1) In the chondrocyte model of OA, glycolytic activity and glycolytic rate significantly increased, accompanied by upregulation of HK2 expression. Overexpression of HK2 promotes catabolism and inflammatory responses while inhibiting anabolic pathways in chondrocytes. 2) Inhibition of HK2 has potent anti-inflammatory and anti-catabolic properties as it increases the expression of HMGA2. 3) PEMF has anti-inflammatory, anti-catabolic, and anti-glycolytic activities, and could alleviate the OA phenotype by modulating the HK2/HMGA2 signaling axis. 4) The HK2 inhibitor, LND, in combination with PEMF, more effectively ameliorated cartilage degeneration and inhibited subchondral bone reconstruction in OA.

## Supplementary Material

Supplementary figures and table.

## Abbreviations

AAV: adeno-associated virus
DMM: destabilization of the medial meniscus
FLS: fibroblast-like synovial
H&E: hematoxylin and Eosin
LA: lactic acid
NSAID: non-steroidal anti-inflammatory drugs
OCR: oxygen consumption rate
ECAR: extracellular acidification rate
PEMF: pulsed electromagnetic field
RA: rheumatoid arthritis
SPF: Specific pathogen-free
TCA: tricarboxylic acid
ECM: extracellular matrix
HMGA2: high-mobility group AT-hook 2
OA: osteoarthritis
HK2: hexokinase 2
LND: lonidamine
OXPHOS: oxidative phosphorylation
