# Supplementary material for: Hexokinase 2 Suppression Alleviates the Catabolic Properties in Osteoarthritis via HMGA2 and Contributes to Pulsed Electromagnetic Field-mediated Cartilage Protection
Source: Int J Biol Sci. 2025 Jan 27;21(4):1459–77. doi: 10.7150/ijbs.101597 (PMC11844298; doi:10.7150/ijbs.101597)
Supplement: Supplementary file 1 — Supplementary figures and table. [file ijbsv21p1459s1.pdf]

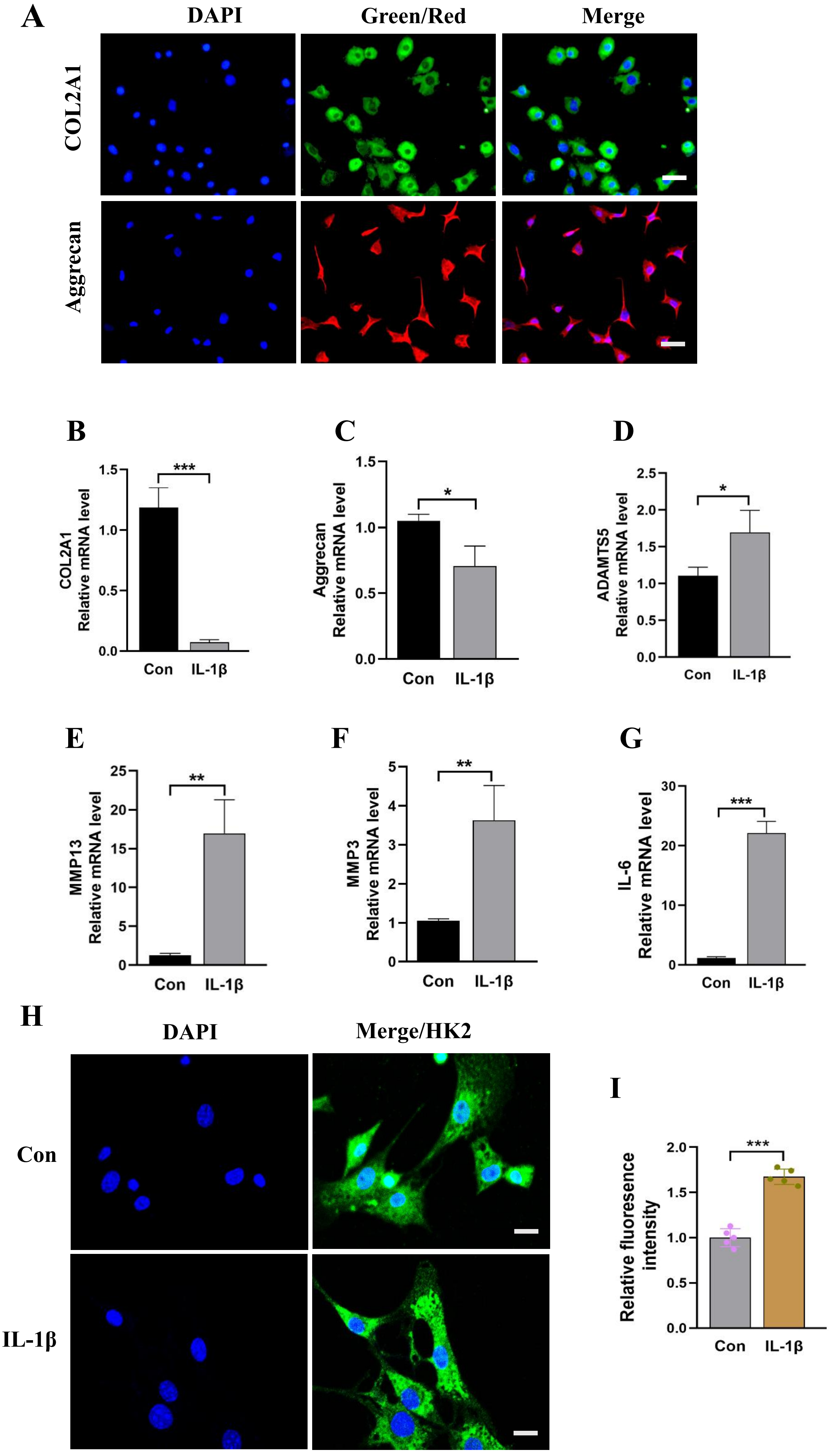

Figure S1. Damaged human knee cartilage and inflammatory stimulation of chondrocytes show high expression of HK2. (A) Analysis of COL2A1 and Aggrecan position and expression using immunofluorescence (red represents Aggrecan, green represents COL2A1, nuclei counterstained with DAPI, scale bars, 100μm). (B-G) Relative COL2A1, Aggrecan, MMP3, MMP13, ADAMTS5 and IL-6 mRNA expression from RT-qPCR. *$p < 0.05$, **$p < 0.01$, ***$p < 0.001$. (H-I) Immunofluorescence was used to detect HK2 positive cells (red represents HK2, nuclei counterstained with DAPI, scale bars, 100μm).

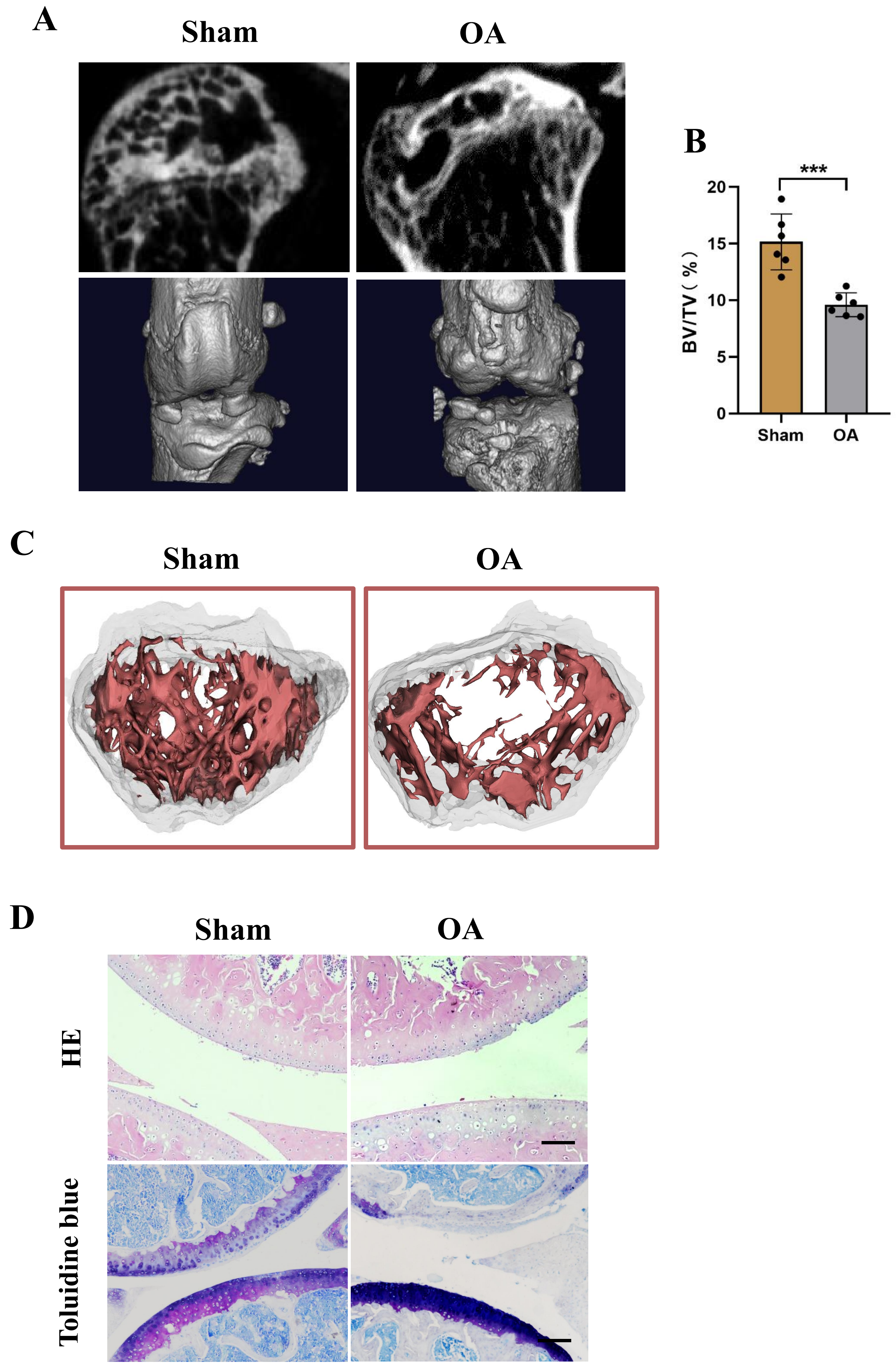

Figure S2. Increased HK2 activity and expression in the DMM model. (A) Changes in bone microstructure changes in the knee joints of OA and Sham mice were analyzed using micro-CT. (B) The graph represents the percentage of BV/TV in the Sham and OA groups. $***p < 0.001$. (C) Three-dimensional imaging reconstruction of the knee joint was performed using Mimics Innovation Suite 21 software. (D) The knee joint tissues of the mice were analyzed using H&E staining and Toluidine blue staining (scale bars, 100μm).

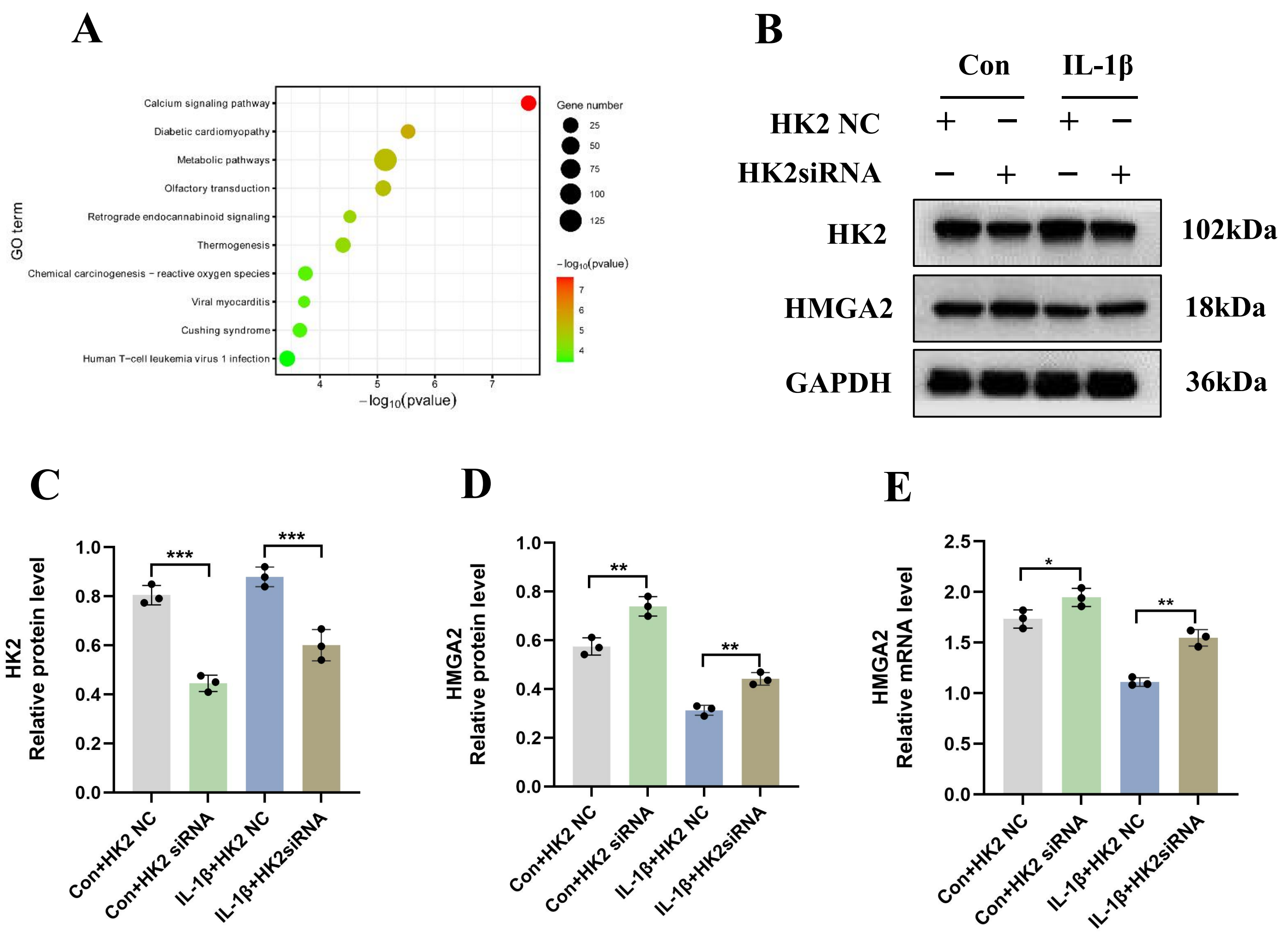

Figure S3. HK2 promotes catabolic activity in OA chondrocytes by inhibiting HMGA2. (A) Pathway enrichment analysis revealed that differentially expressed genes were enriched in metabolic-related pathways. (B-C) Western blot was performed to evaluate HMGA2 protein expression after HK2 suppression in OA chondrocytes, with densitometric quantification of the Western blot results. $**p < 0.01$, $***p < 0.001$. (D) Relative HMGA2 mRNA expression from RT-qPCR. $**p < 0.01$, $***p < 0.001$.

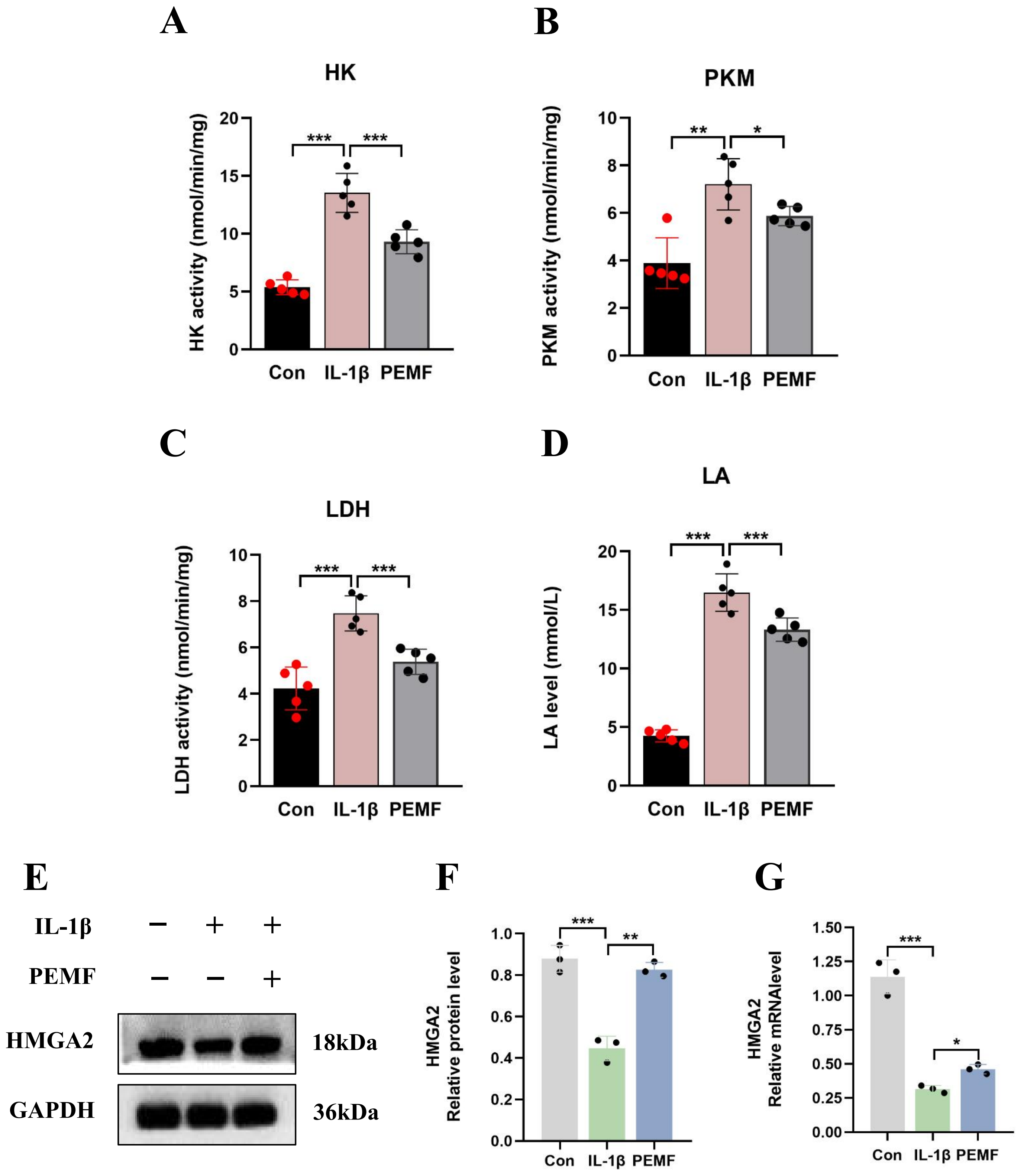

Figure S4. The impact of PEMF on the synthesis, catabolism, and glycolysis activity of OA chondrocytes. (A-D) The enzyme activities of LDH, PKM, and HK, as well as lactic acid (LA) levels, were measured using commercial assay kits after PEMF treatment of IL-1β-induced mouse chondrocytes. *$p < 0.05$, **$p < 0.01$, ***$p < 0.001$. (E-G) After PEMF treatment of IL-1β-induced mice chondrocytes, mRNA and protein levels of HMGA2 were assessed using Western blot and qRT-PCR analysis. *$p < 0.05$, **$p < 0.01$, ***$p < 0.001$.

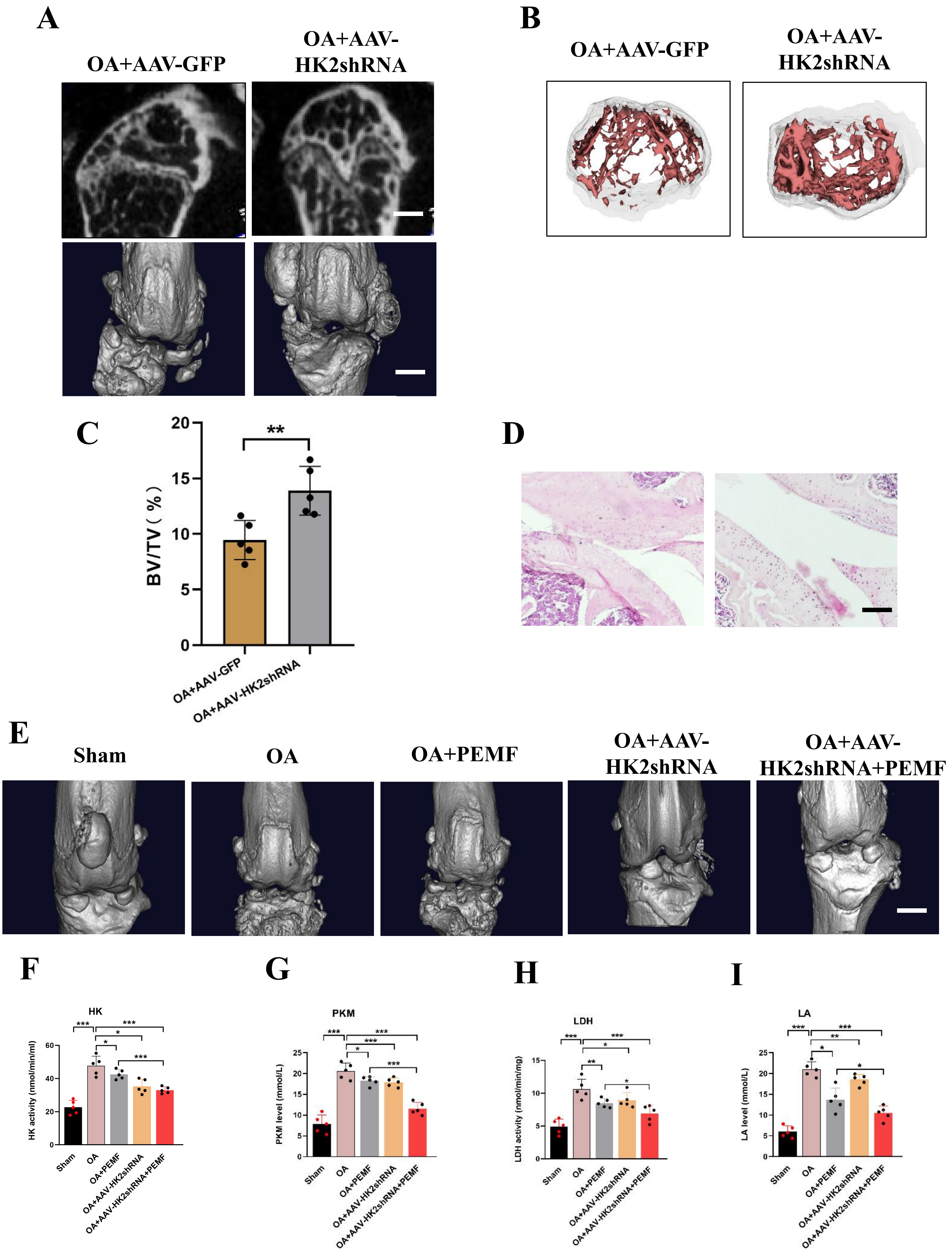

Figure S5. Inhibiting HK2 promotes PEMF to improve joint cartilage damage caused by OA. (A) Cartilage samples were isolated from mouse knee joints and analyzed using micro-CT. Osteophyte formation was observed in the OA+GFP-AAV and OA+AAV-HK2shRNA groups. (B) 3D reconstruction of the knee joint was performed using Mimics Innovation Suite 21 software. (C) The graph shows the percentage of BV/TV (bone volume/total volume) calculated from micro-CT data in the OA+GFP-AAV and OA+AAV-HK2shRNA groups. (D) Safranin O-fast green staining was performed to analyze knee joint tissues in OA mice (scale bars, 100μm). (E) Osteophyte formation was confirmed in each group using histological examination. (F-I) The enzyme activities of LDH, PKM, and HK, as well as lactic acid (LA) levels, were measured using commercial assay kits following PEMF intervention and AAV injection. *$p < 0.05$, **$p < 0.01$, ***$p < 0.001$.

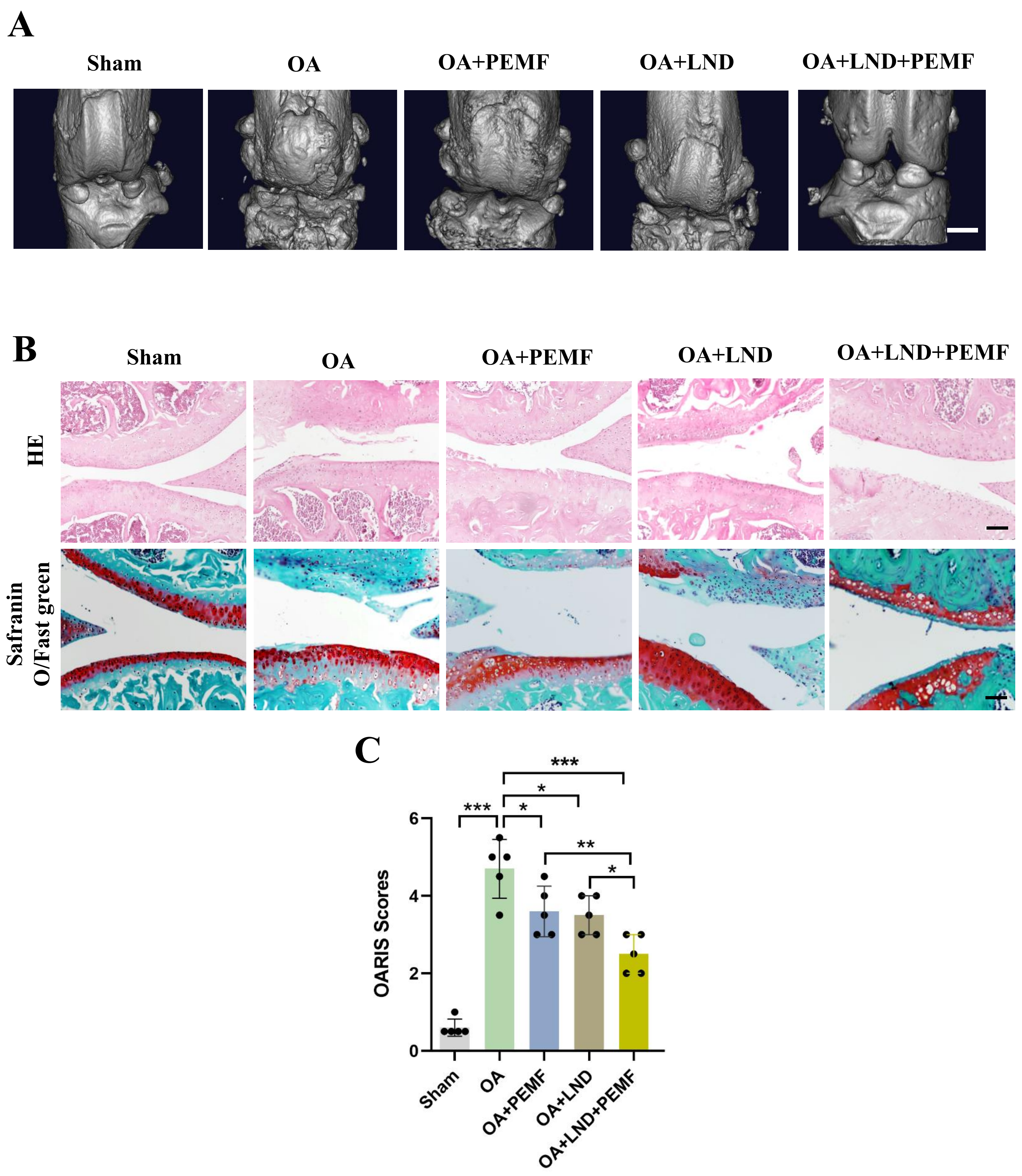

Figure S6. HK2 inhibitor combined with PEMF alleviates cartilage damage caused by OA. (A) Cartilage samples were isolated after PEMF intervention and HK2 inhibitor Lonidamine (LND) injection, and analyzed using micro-CT to assess cartilage structure in each group. (B) The OA mice knee joint tissues were analyzed using Safranin O-fast green staining and H&E staining (scale bars = 100μm). (C) Cartilage damage was evaluated using the Osteoarthritis Research Society International (OARSI) scoring system. *$p < 0.05$, **$p < 0.01$, ***$p < 0.001$.

Table 1

Table 1 Primer sequences

Gene	Sequence	Primer sequences (5'-3')	Length
HK2	Forward	AAGGGGCTAGGAGCTACCAC	20
	Reverse	TTACTCGGAGCACACGGAAG	20
GAPDH	Forward	CGTGTTCCTACCCCCAATGT	19
	Reverse	TGTCATCATACTTGGCAGGTTTCT	24
Aggrecan	Forward	CCTGCTACTTCATCGACCCC	20
	Reverse	AGATGCTGTTGACTCGAACCT	21
HMGA2	Forward	GAGCCCTCTCCTAAGAGACCC	21
	Reverse	TTGGCCGTTTTTCTCCAATGG	21
MMP13	Forward	CTTCTTCTTGTTGAGCTGGACTC	23
	Reverse	CTGTGGAGGTCACTGTAGACT	21
MMP3	Forward	ACATGGAGACTTTGTCCCTTTTG	23
	Reverse	TTGGCTGAGTGGTAGAGTCCC	21
IL-6	Forward	CCAAGAGGTGAGTGCTTCCC	20
	Reverse	CTGTTGTTCAGACTCTCTCCCT	22
COL2A1	Forward	CAGGATGCCCGAAAATTAGGG	21
	Reverse	ACCACGATCACCTCTGGGT	19
ADAMTS5	Forward	GGAGCGAGGCCATTTACAAC	20
	Reverse	CGTAGACAAGGTAGCCCACTTT	22
